# Case report: Association between PTEN-gene variant and an aggressive case of multiple dAVFs

**DOI:** 10.3389/fneur.2024.1347289

**Published:** 2024-04-08

**Authors:** Glaucia Suzanna Jong-A-Liem, Talita Helena Martins Sarti, Mariusi Glasenapp dos Santos, Luciano Marcus Tirotti Giacon, Raphael Wuo-Silva, Alex Machado Baeta, José Maria de Campos Filho, Feres Chaddad-Neto

**Affiliations:** ^1^Department of Neurology and Neurosurgery, Universidade Federal de São Paulo, São Paulo, SP, Brazil; ^2^Department of Neurosurgery, Hospital Beneficência Portuguesa de São Paulo, São Paulo, SP, Brazil; ^3^Department of Neurosurgery, Universidade Federal de Santa Maria, Santa Maria, RS, Brazil; ^4^Department of Neuroradiology, Hospital Beneficência Portuguesa de São Paulo, São Paulo, SP, Brazil; ^5^Department of Neurology, Hospital Beneficência Portuguesa de São Paulo, São Paulo, SP, Brazil

**Keywords:** pseudotumor cerebri, gene variant, AV fistulas, dural arteriovenous fistula (DAVF), PTEN gene mutation

## Abstract

**Introduction:**

Mutations of the phosphatase and tensin homolog (PTEN) gene have been associated with a spectrum of disorders called PTEN hamartoma tumor syndrome, which predisposes the individual to develop various types of tumors and vascular anomalies. Its phenotypic spectrum includes Cowden syndrome (CS), Bannayan–Riley–Ruvalcaba syndrome (BRRS), Proteus syndrome, autism spectrum disorders (ASD), some sporadic cancers, Lhermitte–Duclos disease (LDD), and various types of associated vascular anomalies.

**Clinical presentation:**

A previously healthy 27-year-old woman was experiencing visual scintillating scotomas and mild chronic headaches for the past 2 years. The initial computed tomographic (CT) and magnetic resonance imaging (MRI) scans did not reveal any abnormalities, but the possibility of pseudotumor cerebri was considered. Furthermore, a cerebral angiogram showed a posterior fossa dural arteriovenous fistula (dAVF), which was initially treated through embolization. However, in spite of proper treatment, this patient experienced multiple recurrent dAVFs in different locations, requiring multiple embolizations and surgeries. Despite exhibiting altered cerebral perfusion and hemodynamics, the patient did not display any significant symptoms until she experienced a sudden stroke resulting from deep venous thrombosis, which was not associated with any medical procedures or medication use. A comprehensive analysis was performed due to the aggressive nature of the dAVFs. Surprisingly, exome sequencing of a blood sample revealed a PTEN gene variant in chromosome 10, indicative of Cowden syndrome. However, no tumors or other vascular lesions were detected in other systems that would constitute Cowden syndrome.

**Conclusion:**

The rapid formation of multiple and complex dAVFs, coupled with not meeting the criteria for any other PTEN-related syndrome, unequivocally leads to the presentation of a novel phenotype of the PTEN germline variant.

## 1 Introduction

The phosphatase and tensin homolog (PTEN) gene is a tumor suppressor gene that inhibits the PI3K/AKT/mTOR and vascular endothelial growth factor (VEGF) signaling pathways, which control cell growth, migration, apoptosis, and angiogenesis (1). Mutations of this gene have been associated with a spectrum of disorders called PTEN hamartoma tumor syndrome and predisposes the individual with the syndrome to develop various types of tumors and vascular anomalies. As this gene is expressed in almost all tissues and cell types, this condition usually involves multiple systems and, therefore, neurological anomalies are quite common.

These PTEN germline mutations are very rare, and their phenotypic spectrum includes Cowden syndrome (CS), Bannayan–Riley–Ruvalcaba syndrome (BRRS), Proteus syndrome, autism spectrum disorders (ASD), some sporadic cancers, Lhermitte–Duclos disease (LDD), and various types of vascular anomalies (2).

This case report, therefore, presents a novel phenotypic expression of the PTEN gene in a previously healthy adult with multiple and complex multiple dural arteriovenous fistulas (dAVFs).

## 2 Clinical presentation

A 27-year-old female lawyer was experiencing visual scintillating scotomas and subtle chronic headaches for the last 2 years. The patient had no comorbidities, no family history of cancer syndromes, and did not use continuous medication. An ophthalmologic investigation revealed her condition to be bilateral papilledema and the patient was referred to a neurologist. The initial computed tomographic (CT) and magnetic resonance imaging (MRI) scans were normal and raised the possibility of pseudotumor cerebri, which required supplemental analysis with digital subtracted angiography (DSA). In this examination, a dural arteriovenous fistula (dAVF) was detected in the posterior fossa, which was successfully embolized, resulting in occlusion without complications. The patient was discharged with no acquired deficit (mRS 1).

**Figure 1:**
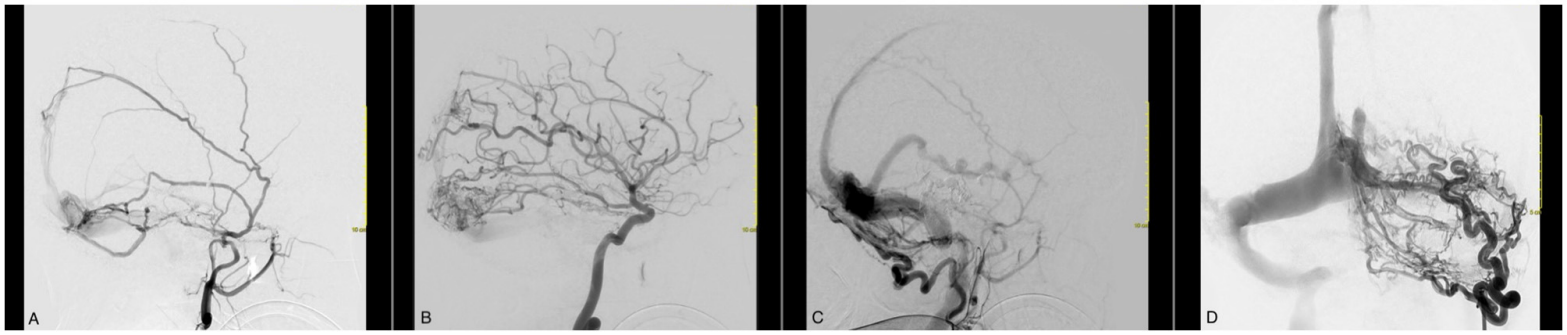
Cerebral angiography with a selective left external carotid artery (ECA) catheterization evidencing an AVF between branches of the external carotid artery and the superficial venous system. Venous congestion and reflux are evident in the Labbe vein and up until the posterior third of the superior sagittal sinus. **(A)** The meningeal branches of ECA in fistulous communication with the superior sagittal sinus and transverse sinus. **(B)** The cortical branches of the ICA in fistulous communication with the superior sagittal and transverse sinus. **(C, D)** A very enlarged occipital artery with multiple branches in fistulous communication with the transverse and sigmoid sinus.

After 1 year, the headaches reoccurred, and the patient was readmitted. In DSA, a new and highly complex dAVF was detected in the supratentorial region, affecting meningeal branches of the external carotid artery, the meningohypophyseal trunk, the ascending pharyngeal and occipital arteries, and both transverse sinuses near the Torcula and superficial cortical veins (Figure 1). Therefore, she underwent a new session of embolization without any complications and was discharged without headaches (mRS 1).

Again, 1 month later, she was readmitted for the third time due to the emergence of a new symptom: tinnitus. A new DSA procedure revealed the presence of a new cortical dAVF in the patient, which was treated with another session of embolization without any complications. The patient was discharged and recovered from the tinnitus but continued to experience a mild headache. As the dAVF pattern changed and symptoms worsened over time, the patient underwent a fourth session of embolization after 3 months and a fifth session after 6 months for the same reasons.

**Figure 2:**
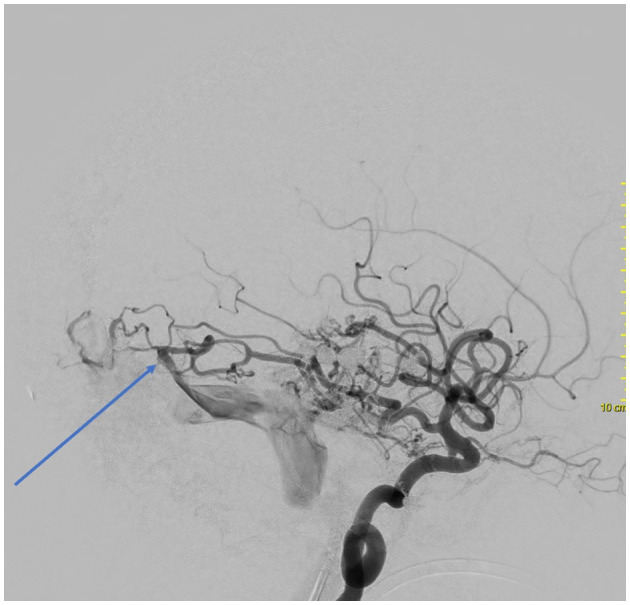
Cerebral angiography with a selective left internal carotid artery catheterization evidencing an AVF between branches of the posterior cerebral artery and the Labbe vein.

Following the completion of the fifth session, the magnetic resonance angiography (MRA) analysis revealed the continued presence of multiple and intricate dAVFs. These dAVFs were supplied by arteries stemming from the right middle meningeal artery, right internal maxillary artery, and occipital artery (Figure 2). Additionally, these dAVFs were directly linked to the right transverse, sigmoid sinus, straight sinus, superior petrosal sinus, and superior sagittal sinus, all of which showed arterial flow. Furthermore, the deep venous drainage, medullary, and cortical veins exhibited signs of venous congestion and indirect indications of brain swelling.

Therefore, the patient underwent a ventriculoperitoneal (VP) shunt with an initial threshold of 150 mmHg. The surgery was uneventful, and the patient was discharged with minor symptoms.

The patient suffered a generalized seizure and fell into a coma during a routine neuroimaging check-up 2 months later. New CT scans revealed a small infarct in the left frontal region with indications of hemorrhagic transformation. MR angiography and venography tests showed tortuous and partial thrombosis in the following veins: the right basal vein of Rosenthal, the sylvian superficial vein, the frontobasal vein, and the right internal jugular bulb. Despite the VP shunt being in the correct position, there were still signs of increased intracranial pressure (ICP).

The patient was kept under sedation for neuroprotection and presented with multiple neurological complications secondary to the mass cerebral venous thrombosis. Despite prolonged periods of anticoagulation therapy, she remained in a comatose condition. A new MRI evidenced the persistence of extensive brain swelling and ischemic parenchymal injury. Given the aggressiveness of the dAVFs and the poor outcome, the patient underwent rheumatologic, immunologic, hematologic, and genetic investigations. The first of these investigations was unremarkable. However, the genetic exome sequencing of her blood sample revealed a pathological variant in the PTEN gene compatible to Cowden syndrome. The patient underwent a full-body screening, which did not reveal any tumors or other vascular lesions in other systems.

The patient's family provided informed consent to publish this case, which was also authorized by the hospital's ethics committee.

## 3 Discussion

The most common neurological phenotypic expressions associated with the PTEN germline mutation manifest as follows.

Macrocephaly is a common finding (80%) in CS and happens to be 100% present in BRRS (3, 4). Lynch et al. studied BRRS in six children with extreme macrocephaly. Four parents of these children also carried the PTEN mutation and asymptomatic extreme macrocephaly as well (3). Hence, we affirm that macrocephaly of idiopathic cause can be an early marker for PTEN mutation-linked disorders (5). Developmental delay is also typical and associated with the macrocephalic finding. Children and adults in these cases have presented a lower intelligence quotient (IQ) score. Objectively, this intellectual disability, measured with an IQ score of 75, is also a minor criterion for the diagnosis of CS (6). ASD, remarkably in association with macrocephaly, has also been linked to PTEN mutation. One in five patients with ASD-macrocephalic have been diagnosed with a PTEN mutation. In these ASD cases, macrocephaly seems to be more severe compared to the non-PTEN-mutated group (7). Individuals with PTEN-related ASD also show anatomical differences, with an increase in cortical white matter and a distinctive neurocognitive and behavioral phenotype, including delayed language development, poor working memory and processing speed, and adaptive and sensory abnormalities (8, 9). Epilepsy has been mentioned in some cases (4, 10, 11) but is not as common in this population.

Lhermitte–Duclos disease (LDD) is the most common brain tumor that develops in adult CS patients. Once identified and confirmed, it is considered a pathognomonic finding for CS (6). This benign lesion occurs in around 35% of the CS patients, and only ~250 cases have been published (12). On T2-MRI, we identify a classic alternating pattern of high and low signal, giving it a striated appearance that is said to resemble “tiger stripes.” This tigroid appearance on T2 imaging distinguishes LDD from other more common primary cerebellar gliomas that destroy the delicate folial pattern of the cerebellum (13). These lesions are benign and progress through a slow growth. Surgical treatment is only required in symptomatic cases (12, 14). In the NIH Genetic Testing Registry, PTEN mutations have also been linked to the appearance of familial meningioma and other predisposing genes. There have been cases of CS associated with meningioma (10). However, whether the meningioma is genuinely associated with the PTEN mutation or is an incidental finding is unclear (15).

Asymptomatic cavernous malformations (cavernoma or cavernous angioma) and developmental venous anomalies (venous angioma) are common, and many cases were reported with these conditions in patients with CS (10). One case of a giant ruptured fusiform middle cerebral artery aneurysm was also reported in a CS patient; however, the PTEN gene mutation was not confirmed because the patient and his family did not consent to genetic testing (16). Two cases of spinal AVF associated with LDD have been reported. Rupture or steal phenomena of the AVF in a cervical location can have devastating clinical consequences. Both cases were very similar in feeding and drainage patterns and could represent a different phenotype (17–19). Non-neurological AVM has been reported in many CS and BRRS cases (20). Real intracranial AVM has not been reported yet. A French CS multicenter study found six patients (in a series of 20) harboring vascular malformation (10). Therefore, the association of PTEN mutations and vascular anomalies is consistent with the growing evidence that PTEN modulates angiogenesis (20).

The literature review for the present study revealed that only a limited number of cases identified a correlation between the PTEN variant and dAVFs. These few cases exhibited the syndromes mentioned earlier. An adult with classic CS experienced a generalized seizure caused by bleeding from multiple dural and epidural high-flow AVFs. This patient underwent five sessions of embolization uneventfully but unfortunately passed away due to severe status epilepticus (21). Another study reported that a 14-year-old with BRRS, who manifested with diplopia and papilledema, was diagnosed with pseudotumor cerebri, similar to the reported case in our study. Despite the signs of increased ICP, no MRI or MR angiogram abnormalities were noted. Because the patient in our study worsened over time, new studies identified dAVF connecting meningeal branches of the middle meningeal artery to the transverse sinus bilaterally. She underwent two sessions of embolization and had a good recovery (22). Acquired dAVF has also been reported in CS in a postop of a brainstem cavernoma. In this case, the manipulated dura underwent neovascularization, creating anomalous communications between dural arteries and cerebellar cortical veins. This patient was reapproached to treat the dAVF. This case suggests that postoperative angiogenesis may cause AVF in patients with CS (23). Determining whether PTEN insufficiency alone or other molecular factors in the PI3K/Akt/VEGF signaling contribute to these angiogenic formations is difficult (24).

Our case, different from the ones mentioned in the literature, did not meet the criteria for any PTEN gene syndrome. Whole-body screening did not identify any cutaneous or visceral lesions, which would fulfill the criteria for CS. She was normocephalic and had no history of developmental delay. Because she was a lawyer, we deduce that the patient previously had an average IQ. Additionally, this case has a unique presentation of rapidly formed multiple and new dAVFs. Despite compromised venous drainage, venous congestion, and chronic perfusion shortages, the patient remained relatively asymptomatic. She only progressed to a more severe neurological state when experiencing acute bleeding secondary to venous thrombosis. Interestingly, her cerebral perfusion adapted rapidly to the collaterals and responded highly to hemodynamic changes after every embolization. This complex formation of dAVF corroborates with the suspicion of physiopathology that PTEN modulates angiogenesis.

It is important to emphasize that this mutation is relatively rare (25).

Of the few cases previously published associated with dAVF, typical PTEN syndromic patients were involved. Hence, we advocate the novelty of this case: (1) Despite the PTEN variant, the patient did not fulfill the criteria for any syndrome; and (2) the rapid and aggressive formation of new dAVFs is not typical. More studies are necessary to understand how to manage patients who present with such dAVFs.

## 4 Conclusion

The previously reported dAVF cases with PTEN variant were linked to syndromic PTEN mutations. We describe a case of a previously healthy adult with multiple and complex dAVFs as a new phenotype.

## Data availability statement

The datasets presented in this article are not readily available because of ethical and privacy restrictions. Requests to access the datasets should be directed to the corresponding author.

## Ethics statement

The studies involving humans were approved by the Ethics and Research Committee of the Federal University of São Paulo. The studies were conducted in accordance with the local legislation and institutional requirements. The participants provided their written informed consent to participate in this study. Written informed consent was obtained from the individual(s) for the publication of any potentially identifiable images or data included in this article.

## Author contributions

GJ-A-L: Conceptualization, Investigation, Writing – original draft, Writing – review & editing. TS: Formal analysis, Investigation, Writing – review & editing. MS: Data curation, Methodology, Writing – review & editing. LG: Data curation, Methodology, Writing – review & editing. RW-S: Writing – review & editing. AB: Data curation, Investigation, Writing – review & editing. JC: Resources, Validation, Writing – review & editing. FC-N: Project administration, Supervision, Validation, Writing – review & editing.

## References

[1] Plamper M Gohlke B Woelfle J. PTEN hamartoma tumor syndrome in childhood and adolescence-a comprehensive review and presentation of the German pediatric guideline. Mol Cell Pediatr. (2022) 9:3. 10.1186/s40348-022-00135-1 35187600 PMC8859017

[2] Tan WH Baris HN Burrows PE Robson CD Alomari AI Mulliken JB. The spectrum of vascular anomalies in patients with PTEN mutations: implications for diagnosis and management. J Med Genet. (2007) 44:594–602. 10.1136/jmg.2007.048934 17526801 PMC2597949

[3] Lynch NE Lynch SA McMenamin J Webb D. Bannanyan-Riley-Ruvalcaba syndrome: a cause of extreme macrocephaly and neurodevelopmental delay. Arch Dis Child. (2009) 94:553–4. 10.1136/adc.2008.155663 19321504

[4] Hendriks YMC Verhallen JTCM van der Smagt JJ Kant SG Hilhorst Y Hoefsloot L. Bannanyan-Riley-Ruvalcaba syndrome: further delineation of the phenotype and management of PTEN mutation-positive cases. Fam Cancer. (2003) 2:79–85. 10.1023/A:1025713815924 14574156

[5] Starink TM van der Veen JP Arwert F de Waal LP de Lange GG Gille JJ. The Cowden syndrome: a clinical and genetic study in 21 patients. Clin Genet. (1986) 29:222–33. 10.1111/j.1399-0004.1986.tb00816.x 3698331

[6] Pilarski R Burt R Kolman W Pho L Shannon KM Swisher E. Cowden syndrome and the PTEN hamartoma tumor syndrome: systematic review and revised diagnostic. J Natl Cancer Inst. (2013) 105:1607–16. 10.1093/jnci/djt277 24136893

[7] Mester JL Tilot AK Rybicki LA Frazier TW Eng C. Analysis of prevalence and degree of macrocephaly in patients with germline PTEN mutations and of brain weight in Pten knock-in murine model. Eur J Hum Genet. (2011) 19:763–8. 10.1038/ejhg.2011.20 21343951 PMC3137495

[8] Busch RM Srivastava S Hogue O Frazier TW Klaas P Hardan A. Developmental synaptopathies consortium. Transl Psychiatry. (2019) 9:253. 10.1038/s41398-019-0588-1 31594918 PMC6783427

[9] Frazier TW Embacher R Tilot AK Koenig K Mester J Eng C. Molecular and phenotypic abnormalities in individuals with germline heterozygous PTEN mutations and autism. Mol Psychiatry. (2015) 20:1132–8. 10.1038/mp.2014.125 25288137 PMC4388743

[10] Lok C Viseux V Francoise M Richard MA Gondry-Jouet C Deramond H. Brain magnetic resonance imaging in patients with Cowden syndrome. Medicine. (2005) 84:129–36. 10.1097/01.md.0000158792.24888.d2 15758842

[11] Prats-Sánchez LA Hervás-García JV Becerra JL Lozano M Castaño C Munuera J. Multiple intracranial arteriovenous fistulas in Cowden Syndrome. J Stroke Cerebrovasc Dis. (2016) 25:e93–4. 10.1016/j.jstrokecerebrovasdis.2016.03.048 27105569

[12] Khandput U Huntoon K Smith-Cohn M Shaw A Elder JB. Bilateral recurrent dysplastic cerebellar gangliocytoma (Lhermitte-Duclos Disease) in Cowden Syndrome: a case report and literature review. World Neurosurg. (2019) 127:319–25. 10.1016/j.wneu.2019.03.131 30905649

[13] Zhang HW Zhang YQ Liu XL Mo YQ Lei Y Lin F. MR imaging features of Lhermitte-Duclos disease: case reports and literature review. Medicine. (2022) 101:e28667. 10.1097/MD.0000000000028667 35089210 PMC8797601

[14] Joo G Doumanian J. Radiographic findings of dysplastic cerebellar gangliocytoma (Lhermitte-Duclos Disease) in a woman with Cowden Syndrome: a case study and literature review. J Radiol Case Rep. (2020) 14:1–6. 10.3941/jrcr.v14i3.3814 PMC7535995 33082915

[15] Yehia L Eng C Adam MP Feldman J Mirzaa GM Pagon RA. PTEN Hamartoma Tumor Syndrome. In: Bean LJH Gripp KW Amemiya A, editors. GeneReviews. Seattle, WA: University of Washington, Seattle (2001). 20301661

[16] Toh K Suzuki K Miyaoka R Kitagawa T Saito T Nakano Y. Giant cerebral aneurysm in a patient with Cowden syndrome treated with surgical clipping. World Neurosurg. (2019) 126:336–40. 10.1016/j.wneu.2019.02.245 30904793

[17] Almubarak AO Haq AU Alzahrani I Shail EA. Lhermitte-duclos disease with cervical arteriovenous fistula. J Neurol Surg A Cent Eur Neurosurg. (2019) 80:134–7. 10.1055/s-0038-1670636 30517962

[18] Akiyama Y Ikeda J Ibayashi Y Nonaka T Asai Y Houkin K. Lhermitte-Duclos disease with cervical paraspinal arteriovenous fistula. Neurol Med Chir. (2006) 46:446–9. 10.2176/nmc.46.446 16998279

[19] Bae BG Kim HJ Lee SG Choi JR Hwang C Lee JH. A novel PTEN mutation in a Korean patient with Cowden syndrome and vascular anomalies. Acta Derm Venereol. (2011) 91:88–90. 10.2340/00015555-0994 21103832

[20] Turnbull MM Humeniuk V Stein B Suthers GK. Arteriovenous malformations in Cowden syndrome. J Med Genet. (2005) 42:e50. 10.1136/jmg.2004.030569 16061556 PMC1736111

[21] Srinivasa RN Burrows PE. Dural arteriovenous malformation in a child with Bannayan-Riley-Ruvalcaba Syndrome. Am J Neuroradiol. (2006) 27:1927–9. 17032868 PMC7977879

[22] Moon K Ducruet AF Crowley RW Klas K Bristol R Albuquerquer FC. Complex dural arteriovenous fistula in Bannayan-Riley-Ruvalcaba syndrome. J Neurosurg Pediatr. (2013) 12:87–92. 10.3171/2013.3.PEDS12551 23662932

[23] Sadahiro H Ishihara H Goto H Oka F Shirao S Yoneda H. Postoperative dural arteriovenous fistula in a patient with Cowden disease: a case report. J Stroke Cerebrovasc Dis. (2014) 23:572–5. 10.1016/j.jstrokecerebrovasdis.2013.04.021 23680687

[24] Kar S Samii A Bertalanffy H. PTEN/PI3K/Akt/VEGF signaling and the cross talk to KRIT1, CCM2, and PDCD10 proteins in cerebral cavernous malformations. Neurosurg Rev. (2015) 38:229–36. 10.1007/s10143-014-0597-8 25403688

[25] Busa T Milh M Degardin N Girard N Sigaudy S Longy M. Clinical presentation of PTEN mutations in childhood in the absence of family history of Cowden syndrome. Eur J Paediatr Neurol. (2015) 19:188–92. 10.1016/j.ejpn.2014.11.012 25549896

